# Supplementary material for: Coupled acoustoplasmonic resonators: the role of geometrical symmetries
Source: Nanophotonics. 2025 Jan 3;14(1):75–80. doi: 10.1515/nanoph-2024-0482 (PMC11744449; doi:10.1515/nanoph-2024-0482)
Supplement: Supplementary file 1 — Supplementary Material Details [file j_nanoph-2024-0482_suppl_001.pdf]

Suplementary Material: "Coupled acoustoplasmonic resonators: the role of geometrical symmetries"

Beatriz Castillo López de Larrinzar,[†] Jorge M. García,[†] Chushuang Xiang,[‡] N. D. Lanzillotti-Kimura,[‡] and Antonio García-Martín[*,†]

†*Instituto de Micro y Nanotecnología IMN-CNM, CSIC, CEI UAM+CSIC, Tres Cantos, Spain*

‡*Université Paris-Saclay, CNRS, Centre de Nanosciences et de Nanotechnologies, Paris, France*

E-mail: a.garcia.martin@csic.es

S1. Mechanical response of an isolated bar

When the thermally-excited bar is decoupled from the substrate the system presents one clear mode for a frequency slightly bellow 10GHz. It is clearly shown by the displacement profile of Figure S1 that there is no oscillation in the adjacent bar. Therefore the mode at 9.97 GHz corresponds to the fundamental extensional mode of a single nanobar oscillating in free space.

In Figure. S2, we present the profile of the mode at 10.8GHz for a single bar but now attached to the substrate. In the figure we see the extensional nature of the longitudinal mode, and that the attachment to the substrate produces a deformation in it, that will be

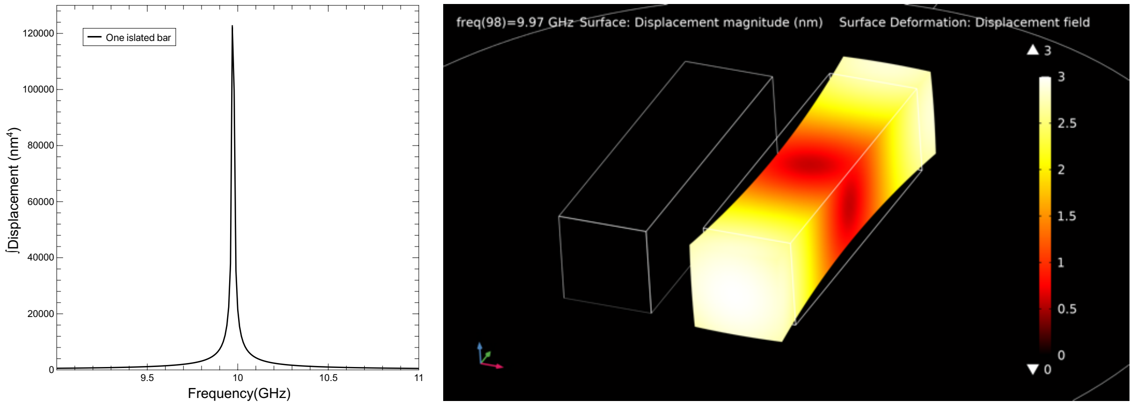

Figure S1: Left: spectral dependence of the integral for the RMS displacement, for two-bar system where the thermally excited bar is detached from the substrate. -Right: displacement magnitude for the mode.

responsible to the mechanical coupling in the two-bar system.

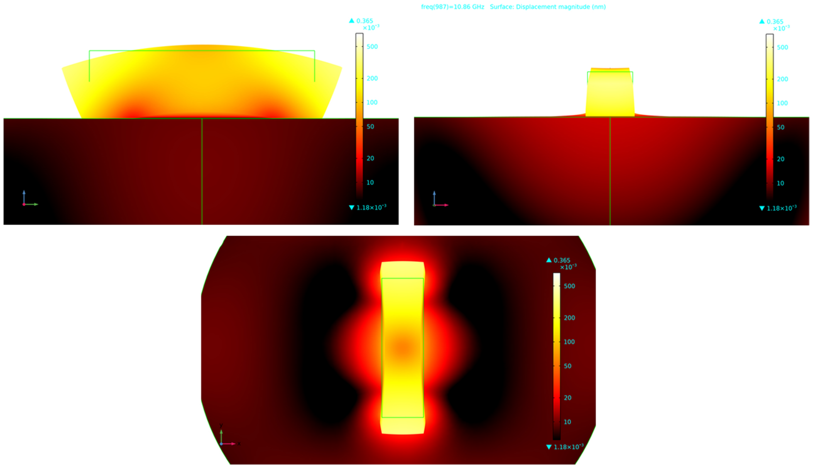

Figure S2: Mode profile for the isolated bar attached to substrate in (top-left) side view, (top-right) front view and (bottom) top view, showing the longitudinal extensional nature of the mode. The displacement originated in the substrate is also visible. The deformation has been exaggerated for a better view.

S2. Dependence of the interaction with the separation distance

The following figures show the low-frequency range of the spectrum of the integral of the RMS displacement for varying distances for the symmetric alignments. They substantiate our findings regarding the interaction through the substrate as responsible for the new modes.

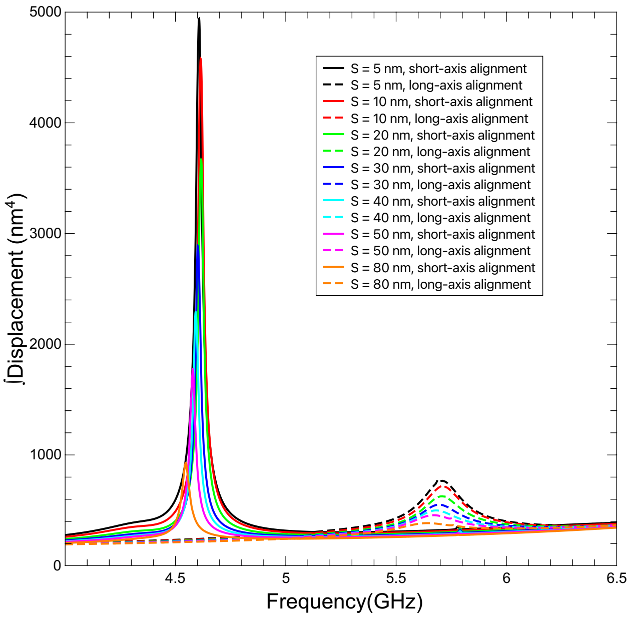

Figure S3: Evolution of the resonance peaks a function of the separation distance S = 5 nm, 10 nm, 20 nm, 30 nm, 40 nm, 50 nm and 80nm for the symmetric alignment, continuous lines are for alignment along the short axis and dashed lines for alignment along the long axis.

For each symmetric configuration, we observe the low-frequency resonance corresponding to each separation distance. The short-axis alignment present a resonant behavior around 4.6 GHz, whereas for long-axis alignment it appears around 5.7 GHz.

Both resonances present a red-shift as the separation is increased. We can appreciate that, even when the bars are 80nm apart, they still interact through the substrate, irrespective of the alignment. For sufficiently big distances those resonances will disappear and the spectrum will present only the high frequency resonance.

S3. Symmetric thermal excitation

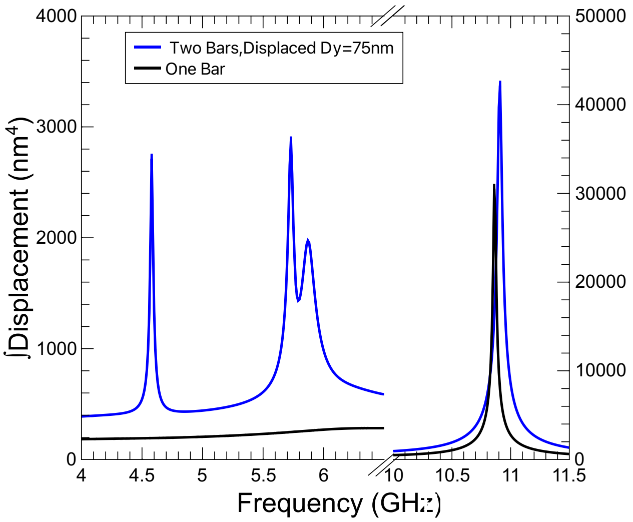

Figure S4: Integral of the RMS displacement as a function of frequency for Dy = 75 nm for a symmetric thermal excitation

The main goal of our work is to present a situation where symmetries are broken, not only in the geometry but also in the excitation. However, an experimental realization where only one bar is excited is challenging. However, the main conclusion of our work is robust, since a simultaneous excitation of the two bars would remove only the "counterphase mode", but the "inphase" mode will survive and be dominant resonance in that case. Therefore there would be still a torsional mode induced by the geometrical broken symmetry, as shown in Figure S4.

S4. Multipolar decomposition of the optical resonances

To provide a more rigorous argument we present in Figure S5 the results of a multipolar decomposition of the induced currents in the structure with broken mirror symmetry for each polarization. As we illuminate the system with a plane wave polarized along one direction

(either long or short axis), if the resonances were not coupled, there would not be any induced dipole in the perpendicular direction. The multipolar decomposition shows that there are induced dipoles, whose ratios are presented in Figure S5.

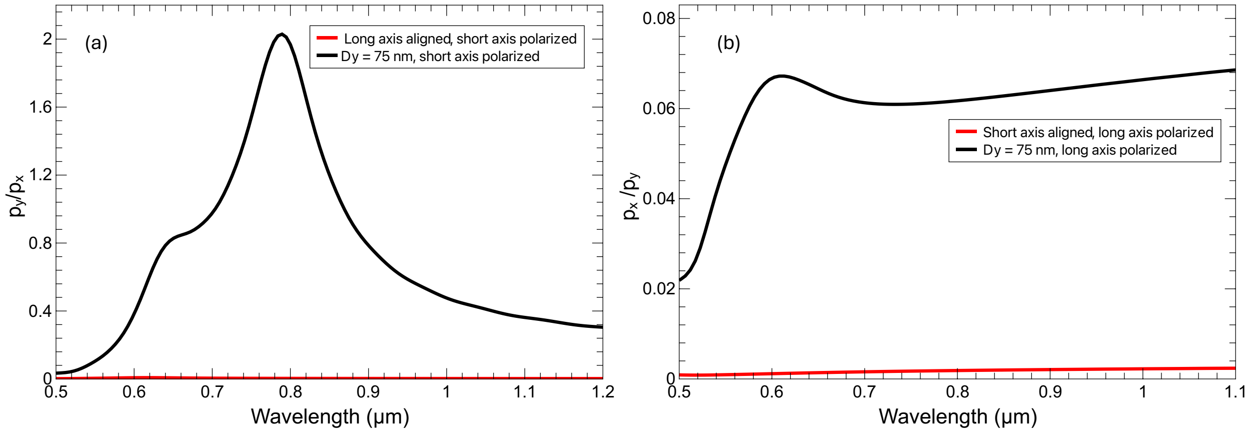

Figure S5: Ratio of the dipoles that appear when we illuminate the system along the (a) short axis of polarization or (b) long axis of polarization.

The black curve in Figure S5(a) represents the ratio p_y/p_x when the system is illuminated along the short axis of polarization, and in Figure S5(b), represents the ratio p_x/p_y when the system is illuminated along the long axis of polarization. As it can be seen, in each situation there is an induced dipole in the axis perpendicular to the polarization axis. In the specific case that we are concerned about, when the incident plane wave is polarized along the long axis, the induced dipole due to the coupling of the bars in the perpendicular direction, p_x, is two orders of magnitude smaller than the dipole that appears in the polarization axis, p_y, and thus its relative weight in the cross-section is far weaker. Red curves for Figure (a) and (b) represent the induced dipole when the bars are aligned along the short (long) axis and the incidence is along the long (short) axis. As expected, there are no induced dipoles (up to the numerical error) in the perpendicular direction as the bars are coupled only in one direction.

S5. Displacement profiles of the mechanical modes, and in the substrate.

Here we present the actual displacement profiles of the mechanical modes sketched in Figures 3 and 4 of the main text.

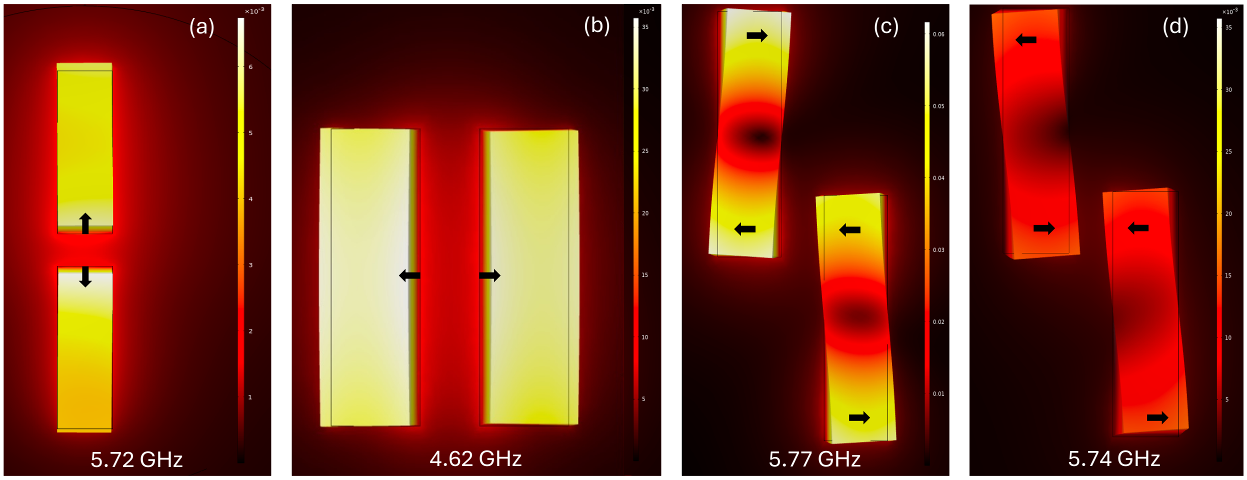

Figure S6: Mechanical modes associated to the resonances when the system is (a) long axis aligned, (b) short axis aligned, (c) and (d) Dy = 75 nm.

In Figure S7 we present the displacement of the substrate for a plane located inside the substrate at (a) 1 nm, (b) 20 nm and (c) 100 nm from the bottom of the nanobars. Additionally, we present in (d) a cross-section in the yz plane for x=0 depicting the asymmetry of the excited wave.

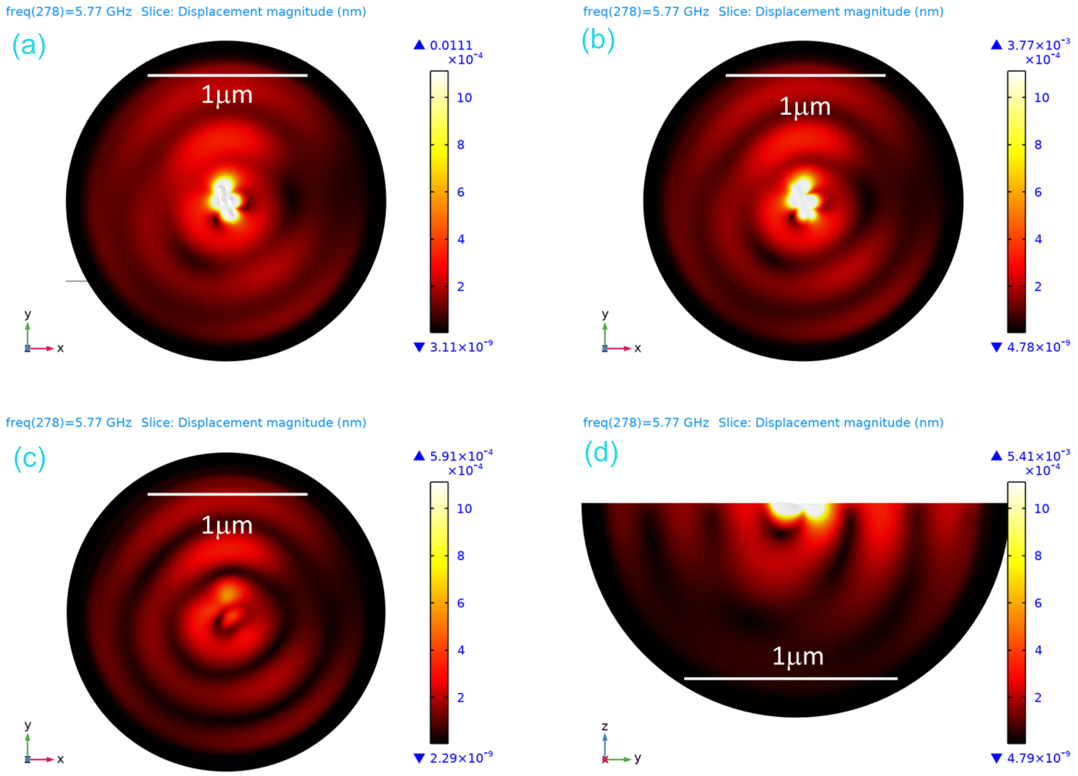

Figure S7: (a) Deformation profile into the substrate at 1nm from the bottom of th nanobars. (b) The same at 20nm. (c) The same at 100nm. (d) Deformation profile at the x=0 plane.
